# Network-based assessment of the selectivity of metabolic drug targets in *Plasmodium falciparum* with respect to human liver metabolism

**DOI:** 10.1186/1752-0509-6-118

**Published:** 2012-08-31

**Authors:** Susanna Bazzani, Andreas Hoppe, Hermann-Georg Holzhütter

**Affiliations:** 1 Institut für Biochemie, Charite-Universitätsmedizin, Reinickendorfer Str. 61, Haus 10, 13347 Berlin

**Keywords:** *Plasmodium falciparum*, Human hepatocyte, Drug targets, Drug selectivity, Genome-scale networks, Reduced fitness, Flux balance analysis

## Abstract

**Background:**

The search for new drug targets for antibiotics against *Plasmodium falciparum*, a major cause of human deaths, is a pressing scientific issue, as multiple resistance strains spread rapidly. Metabolic network-based analyses may help to identify those parasite’s essential enzymes whose homologous counterparts in the human host cells are either absent, non-essential or relatively less essential.

**Results:**

Using the well-curated metabolic networks PlasmoNet of the parasite *Plasmodium falciparum* and HepatoNet1 of the human hepatocyte, the selectivity of 48 experimental antimalarial drug targets was analyzed. Applying *in silico* gene deletions, 24 of these drug targets were found to be perfectly selective, in that they were essential for the parasite but non-essential for the human cell. The selectivity of a subset of enzymes, that were essential in both models, was evaluated with the reduced fitness concept. It was, then, possible to quantify the reduction in functional fitness of the two networks under the progressive inhibition of the same enzymatic activity. Overall, this *in silico* analysis provided a selectivity ranking that was in line with numerous *in vivo* and *in vitro* observations.

**Conclusions:**

Genome-scale models can be useful to depict and quantify the effects of enzymatic inhibitions on the impaired production of biomass components. From the perspective of a host-pathogen metabolic interaction, an estimation of the drug targets-induced consequences can be beneficial for the development of a selective anti-parasitic drug.

## Background

An ideal drug should tackle the disease-causing processes in the most selective way, i.e. with no harm for the healthy cells. To our knowledge, such a perfectly selective drug does not exist. In fact, administrated chemicals can trigger at least secondary consequences (off-target effects) in the organism or in host cells [1]. In case of anti-parasitic drugs, side-effects on the metabolism of host cells may arise from the binding to homologous proteins that share a long evolutionary history with the parasite [2]. To minimize such side-effects, analyses on the performance of multiple networks and the consequences of enzymatic homologous inhibitions may be useful. Ideally, this analysis should be based on detailed mechanistic models of the metabolic networks of the parasite and the host cell pathways [3,4]. However, all the kinetic information needed for the creation of such models is rarely available. Therefore, alternative modeling frameworks, such as Monte Carlo simulations [5] and flux balance analysis (FBA) [6], have been developed for the simulation of the cellular metabolism. These methods allow the simulation of genome-scale networks, do not require kinetic enzymatic knowledge and are suited to test the outcome of gene deletions [6-8].

Genome-scale metabolic models of pathogens are extensively exploited to predict putative drug targets with FBA frameworks [9-11]. Furthermore, recent network-based analyses integrate host and pathogen models to describe more accurately the metabolic interactions and to improve the search of putative drug targets. For example, the topology of automatically inferred networks of *Plasmodium falciparum* and its human host are studied to identify essential enzymes [12]. The metabolic network of *Mycobacterium tubercolosis* is integrated with a human alveolar macrophage to describe three degrees of infection (latent, pulmonary, and meningeal tuberculosis) [13]. Similarly, a metabolic model of *Plasmodium falciparum* is embedded in a red blood cell model to simulate its intra-erythrocytic developmental stage [14]. Furthermore, the selectivity of enzymatic drug targets is already extensively predicted with large-scale metabolic networks of human cancer cells [15]. Thus, it is reasonable to assess the selectivity of enzymatic drug targets in host-pathogen metabolic interactions with genome-scale networks. The aim of this study was the prediction of selective enzymatic inhibitions with genome-scale networks of *Plasmodium falciparum* (PlasmoNet) and the human hepatocyte (HepatoNet1) [14,16]. Although the intra-erythrocytic plasmodial phase is still extensively investigated, the intra-hepatic phase of development is the first infection site and, thus, a promising stage of treatment [17]. To predict feasible metabolic phenotypes, for each model a set of metabolic objectives was assembled with extensive literature search. This set described the cellular composition that is specific to the modeled cell type. Then, computational methods were applied to predict the selectivity of antimalarials in absolute and in relative sense. To predict the enzymatic inhibitions in absolute terms, *in silico* gene deletions of homologous enzymes were performed. Alternatively, the concept of reduced fitness was applied to homologous essential enzymes to assess inhibitions in relative terms. While *in silico* gene deletions predicted scenarios where the enzyme is fully disrupted (e.g. with gene-excision experiments), the second approach better described the possibility of residual enzymatic activity. The latter is more appropriate for the administration of enzymatic inhibitors that only gradually reduce the enzyme activity.

The set of experimental antimalarial targets, that were tested in this work, were a merged and pruned list of published “gold standards”. The merged list contained 96 enzymes, out of which 48 were selected. These enzymes are known to be essential for *Plasmodium falciparum* metabolic homeostasis and growth [14,18,19]. To test the selectivity of these enzymes, *in silico* gene deletions of the enzymatic set were simulated. Twenty-four enzymes were found to be essential only for the parasite and did not compromise the host metabolic performance. Twelve enzymes were essential for both models and were investigated with the concept of reduced fitness [20], to identify which network could be more sensitive to an enzymatic impairment. Then, the fitness-based selectivity score predicted that 10 enzymes out of this last set were more sensitive for the host, as their *in silico* perturbations had a larger impact on the achievement of host metabolic objectives (Table 1).

**Table 1:** Outcome of the simulated drug target inhibitions

**EC**			**HN gene**	**PN gene**		**RF50 HN perturbed**	**RF50 PN perturbed**	
**number**	**Enzyme**	**General reaction**	**deletion**	**deletion**	**RF Score**	**metabolites (deviation %)**	**metabolites (deviation %)**	**Sources**
	Acyl-CoA	Fatty Acid + ATP + CO2 →				Cardiolipin	Sphingomyelin	
6.2.1.3	synthetase	Fatty Acid CoA + AMP + PPi	X	X	1.97	(mitochondrion) (-50%)	(-97.07%)	[17]
	Thymidylate	dUMP + 5,10 Methylene THF ↔						
2.1.1.45	synthase	dTMP + DHF	X	X^0^	1.00	dTTP (-50%)	DNA (nucleus) (-50.31%)	[12,14,17]
4.1.1.23	Orotidine 5P decarboxylase	Orotidine 5P →UMP + CO2	X	X	0.713	UDP-Glucose (-80.57%)	mRNA (nucleus) (-57.44%)	[12,14,17]
2.4.2.10	Orotate phosphoribosyltransferase	Orotidine 5P + PPi ↔	X	X	0.713	UDP-Glucose (-80.57%)	mRNA (nucleus) (-57.44%)	[17]
		Orotate +PRPP						
2.1.3.2	Aspartate carbamoyltransferase	Carbamoyl-P + Aspartate →	X	X^1^	0.713	UDP-Glucose (-80.57%)	mRNA (nucleus) (-57.44%)	[17]
		Carbamoyl-Aspartate + Pi						
3.5.2.3	Dihydroorotase	N-Carbamoyl-Aspartate →	X	X^1^	0.713	UDP-Glucose (-80.57%)	mRNA (nucleus) (-57.44%)	[14,17]
		S-Dihydroorotate + H2O						
4.3.2.2	Adenylosuccinate lyase	Adenylsuccinate →	X	X	0.611	NADPH (-91.23%)	mRNA (nucleus) (-59.25%)	[17]
		Fumarate + AMP						
2.1.2.1	Serine hydroxymethyltransferase	5,10 Methylene THF + Glycine +	X	X^2^	0.514	NADPH (-91.92%)	DNA (nucleus) (-50.31%)	[12,14,17]
		H2O ↔ THF + Serine						
1.5.1.3	Dihydrofolate reductase	THF + NAD(P)H ↔ DHF + NAD(P)	X	X	0.50	dTTP (-50%);	DNA (nucleus) (-50.31%)	[12,14,17]
						Tetrahydrofolate (-50%)		
6.4.1.2	Acetyl-CoA carboxylase	ATP + Acetyl-CoA + HCO3- →	X	X	0.47	Triacylglycerol (+105.11 %)^3^	Protein N6 (lipoyl)lysine	[12,17]
		Malonyl-CoA + ADP + Pi					(apicoplast) (-50%)	
6.3.5.5 & 6.3.4.16	Carbamoyl-P synthetase	2 ATP + Glutamine + HCO3- + H2O →	X	X^1^	0.404	UDP-Glucose (-80.56%);Urea (-61.3%)	mRNA (nucleus) (-57.43%)	[17]
		2 ADP + Pi +Glutamate + Carbamoyl-P						
2.3.1.15	Glycerol 3P acyltransferase	Acyl-CoA + glycerol 3P →	X	X^4^	0.37	Phosphatidyl ethanol amine (-55.77%);	Phosphatidyl choline (-94.53%)	[12]
		CoA + 1-acyl-glycerol 3P				Triacylglycerol (-50%);		
						Phosphatidyl inositol (-50%);		
						Phosphatidyl choline (-50%); Cardiolipin (-50%)		
2.3.1.50	Serine C-palmitoyl transferase	Serine + Palmitoyl-CoA ↔	O	X	*∞*	-	-	[14]
		3-Dehydrosphinganine + CoA + CO2						
		CoA + CO2						
1.17.4.1	Ribonucleotide reductase	dNDP + Ox. Thioredoxin ↔	O	X	*∞*	-	-	[12,14,17]
		NDP + Thioredoxin						
2.3.1.37	5-aminolevulinate synthase	Glycine + Succinyl-CoA ↔	O	X	*∞*	-	-	[12,14,17]
		5-aminolevulinate + CoA + CO2						
2.5.1.6	S-Adenosyl methionine synthase	Methionine + ATP ↔	O	X	*∞*	-	-	[17]
		S-Adenosyl-Methionine + PPi + Pi						
2.7.6.1	Phosphoribosyl pyrophosphate synthase	ATP + Ribose 5P ↔	O	X	*∞*	-	-	[17]
		PRPP + AMP						
2.7.7.15	Choline phosphate citidyl transferase	CTP + Phosphocholine →	O	X	*∞*	-	-	[17]
		PPi + CDP-Choline						
1.15.1.1	Superoxide dismutase	2 O2- + 2 H+ → O2 + H2O2	O	X	*∞*	-	-	[12,14,17]
2.3.1.24	Sphingosine N-Acyl transferase	Acyl-CoA + Sphingosine ↔ CoA + Ceramide	O	X	*∞*	-	-	[14]
1.8.1.7	Glutathione reductase	2 GSH + NADP+ ↔ GSSG + NADPH + H+	O	X	*∞*	-	-	[14,17]
1.8.1.9	Thioredoxin reductase	Thioredoxin + NADP+ ↔ Thioredoxin disulfide + NADPH	O	X	*∞*	-	-	[14,17]
4.2.1.24	Delta aminolevulinate dehydratase	2 5-aminolevulinate → porphobilinogen + 2 H2O	O	X	*∞*	-	-	[12,14,17]
3.3.1.1	S-adenosyl-l-homocysteine hydrolase	S-adenosyl-L-homocysteine + H2O → L-homocysteine + Adenosine	O	X	*∞*	-	-	[12,14,17]
1.10.2.2	mitochondrial Ubiquinone-Cytochrome C reductase	QH2 + 2 ferricytochrome c ↔ Q + 2 ferrocytochrome c + 2 H+	O	X	*∞*	-	-	[12,14,17]
4.2.1.1	Carbonate dehydratase	H2CO3 ↔ CO2 + H2O	O	X	*∞*	-	-	[14]
2.7.8.3	Sphingomyelin synthase	CDP-choline + a ceramide → CMP + sphingomyelin	O	X	*∞*	-	-	[14,17]
1.1.1.27	L-lactate dehydrogenase	(S)-lactate + NAD+ ↔ pyruvate + NADH + H+	O	X	*∞*	-	-	[17]
6.3.2.2	Gamma-glutamylcysteine synthetase	ATP + Glutamate + Cysteine → ADP + Pi + gamma-Glutamyl-cysteine	O	X	*∞*	-	-	[12,14,17]
6.3.4.2	CTP synthase	ATP + UTP + Glutamine + H2O → ADP + Pi + Glutamate + CTP	O	X	*∞*	-	-	[17]
6.3.4.4	Adenylosuccinate synthase	GTP + IMP + Aspartate → GDP + Pi + Adenylosuccinate	O	X	*∞*	-	-	[14], [17]
1.9.3.1	Cytochrome c oxidase	4 ferrocytochrome c + O2 + 4 H+ ↔ 4 ferricytochrome c + 2 H2O	O	X	*∞*	-	-	[17]
2.4.2.1	Purine nucleoside phosphorylase	Inosine + Pi ↔ Ribose 1P + Hypoxanthine	O	X	*∞*	-	-	[17]
6.2.1.1	Acetyl-CoA synthase	ATP + Acetate + CoA →Acetyl-CoA + AMP + PPi	O	X	*∞*	-	-	[17]
2.4.2.8	Hypoxanthine guanine phosphoribosyl transferase	Nicotinate D-ribonucleoside + Pi -→Nicotinate + Ribose 1P	O	X	*∞*	-	-	[12,14,17]
6.3.2.17	Folylpoly glutamate synthase	ATP + tetrahydropteroyl-[gamma-Glu]n + L-glutamate → ADP + phosphate +	O	X	*∞*	-	-	[17]
		tetrahydropteroyl-[gamma-Glu]n+1						
1.1.1.205	IMP dehydrogenase	IMP + NAD + H2O →Xanthosine 5P + NADH	O	•^5^	0	-	-	[12,14,17]
1.6.99.3	NADH dehydrogenase	Acceptor + H^+^+ NADH ↔ Reduced Acceptor + NAD+	O	•^6^	0	-	-	[14]
2.5.1.16	Spermidine synthase	S-Adenosylmethioninamine + Putrescine ↔ 5-Methylthioadenosine + Spermidine	O	•^7^	0	-	-	[14] , [12]
2.7.1.32	Choline kinase	Choline + ATP → Phosphocholine + ADP	O	•^8^	0	-	-	[12,14,17]
3.5.4.4	Adenosine deaminase	Adenosine + H2O ↔ Inosine + NH3	O	•^9^	0	-	-	[12,14,17]
4.1.1.50	S-Adenosyl methionine decarboxylase	S-Adenosylmethionine ↔ Adenosylmethioninamine + CO2	O	•^10^	0	-	-	[12,14,17]
4.1.2.13	Aldolase	Fructose 1,6 PPi ↔ Glycerone P + Glyceraldehyde P	O	•^11^	0	-	-	[12,14,17]
6.3.5.2	GMP syntethase	ATP + Xanthosine-5P + Glutamine + H2O → AMP + PPi + GMP + Glutamate	O	•^12^	0	-	-	[14]
4.1.1.17	Ornithine decarboxylase	L-ornithine → putrescine + CO2	O	•^13^	0	-	-	[12,14]
2.7.1.1	Hexokinase	Glucose + ATP → Glucose 6P + ADP	O	•^14^	0	-	-	[17]
2.1.1.103	Phospho ethanolamine N-methyl transferase	SAM + Ethanolamine-P ↔ SAH + N-Methylethanolamine-P	O	•^15^	0	-	-	[14]
5.3.1.1	Triose phosphate Isomerase	D-glyceraldehyde 3-phosphate ↔ Glycerone phosphate	O	•^16^	0	-	-	[17]

^0^lethal with block of the alternative reaction Q00007, EC 1.5.7.1.

^1^to activate the enzyme in the reference state, the import of dihydroorotate should be blocked.

^2^lethal with block of thetrahydrofolate recharging. Block of *R*01221,*Q*00007,*R*07168,*R*01224,*R*01220,*R*01218.

^3^Triacylglycerol is imported, instead to be exported, to compensate the inhibition consequences ( load value=0.063; RF50 inhibited target flux= -0.0032183).

^4^lethal with external depletion of glycerol, 1,2-diacyl glycerol, sn glycerol 3 phosphate, phosphatidylcholine, phosphatidylserine, phosphatidylethanolamine.

^5^lethal with external depletion of adenosine, adenine, hypoxanthine, inosine.

^6^acceptors of the respiratory electron chain are ubiquinone and cytochrome C (complex III), lethal if also complex III is blocked.

^7^lethal with external depletion of spermidine.

^8^lethal with external depletion of phosphatidylcholine.

^9^lethal with external depletion of 5’-methylthioinosine, xanthine, hypoxanthine, inosine.

^10^lethal with external depletion of spermidine.

^11^off-target effects due to the enzymatic role during host invasion.

^12^lethal with external depletion of guanine and guanosine.

^13^lethal with external depletion of putrescine, spermidine and blocked agmatinase (EC 3.5.1.53), in *Plasmodium bergheii* but not yet charaterized in *Plasmodium flaciparum)*.

^14^topologically not essential, synthetically lethal with inhibition of glucose 6p isomerase (EC 5.3.1.9).

^15^lethal with external depletion of phosphatidylcholine and choline.

^16^off-target effects due to cytoskeleton association of the enzyme.

^17^RF Score = selectivity score predicted with the concept of reduced fitness.

**X** = essential enzyme; **O**=non essential enzyme; ∙=conditional essential enzyme.

**HN** = HepatoNet1; **PN**=PlasmoNet; **RF50**=Reduced Fitness at 50% of enzyme inhibition.

## Results and discussion

The gene-deletion approach divided the set of drug targets in three groups (Table 1): (i) enzymes that are essential in both networks, (ii) enzymes that are essential only in PlasmoNet and (iii) enzymes that are dispensable in both networks. The largest set of experimental drug targets (24 out of 48) was not lethal for the host (due to alternative paths that bypassed the deleted reactions) and essential for the parasite network (at least one biomass flux was not achieved). These enzymes are, thus, predicted to be perfectly selective with respect to the *Plasmodium falciparum* network. Intriguingly, 12 drug targets were identified to be dispensable in both models. When the simulations included the experimental *in vitro* conditions that validated the drug targets, 9/12 enzymes turned out to be essential. This situation was defined as *conditional essentiality*, as the enzyme becomes essential when some substrates are not available in the medium. The remaining 3 glycolytic enzymes were, instead, predicted to be non-essential. This could be due to off-target effects, to extra-metabolic functions or to possible assembly of multi-enzymatic complexes.

### Homologous drug targets essential in both networks

The gene-deletion approach predicted that 12 enzymes are essential in both networks (Table 1), thus indicating a possible metabolic hepatic impairment caused by the inhibition of homologous enzymes.

While this enzymatic set is already experimentally tested, a literature search was done to find any evidence of human cellular toxicity.

It was predicted that only *acyl-CoA syntethase* may be a selective target for antimalarial treatment (selectivity score=1.97). The essentiality of this enzyme is experimentally demonstrated in human lymphoblast-like cells [21].

The fitness approach predicted that *thymidylate synthase* was equally detrimental for both models (selectivity score=1). This enzyme is a possible therapeutic drug target that is efficient against a wide spectrum of human tumors [22,23] but one main drawback is the possibility of its enzymatic over-expression that may confer drug resistance.

*Orotidine-5-phosphate decarboxylase* and *orotate phosphoribosyltransferase* form a multienzymatic complex, but for this analysis the two enzymatic activities were assessed separately. Nevertheless, the results obtained with the fitness approach indicated the same degrees of biomass impairment. The cellular toxicity caused by the inhibition of orotidine-5-phosphate decarboxylase was demonstrated in human leukemia cell lines [24], while orotate phosphoribosyltransferase is an antitumoral target for human gastric carcinoma cells [25]. Equally to the effects caused by these two enzymes, also *aspartate carbamoyltransferase* was predicted to inhibit the same biomass components (Table 1). The essentiality of last enzyme is demonstrated in human hepatocytes [26].

Similarly to the cited enzymes, *dihydroorotase* can also impair the synthesis of the same biomass components. Its essentiality is demonstrated in CCRF-CEM leukemic cells [27].

The reduced fitness approach correctly predicted that the enzymatic inhibition of *acetyl-CoA carboxylase* causes an importing of plasma triglycerides and this *phenomenon* has been experimentally demonstrated *in vitro*[28]. Acetyl-CoA carboxylase is the rate-limiting step of the fatty acid anabolism and it is essential for human breast cancer cells [29].

*Carbamoyl-phosphate synthase* was predicted to be more detrimental for the hepatocyte than acetyl-CoA carboxylase. This enzyme catalyzes the formation of carbamoyl phosphate from carbon dioxide and ammonia (EC 6.3.4.1.6) or glutamine (EC 6.3.5.5). Here, it was not discriminated between the ammonia-donors (NH4^+^ or glutamine) because the inhibition of one reaction was able to be fully compensated by the other. The essentiality of carbamoyl phosphate synthetase (EC 6.3.5.5) was demonstrated in mouse liver [30].

The last enzyme of this set was *glycerol-3-phosphate acyltransferase*, that is essential for human hepatocyte cultures [31].

The gene deletions of this antimalarial drug target set were confirmed by the aforementioned experimental data. Moreover, the reduced fitness approach correctly predicted the specific case that was triggered by the inhibition of acetyl-CoA carboxylase: while in the reference state the triglycerides are part of the maintenance function, thus they are exported into the blood, under the perturbation there was an inversion of the boundary flux and they were instead imported.

**Figure 1:**
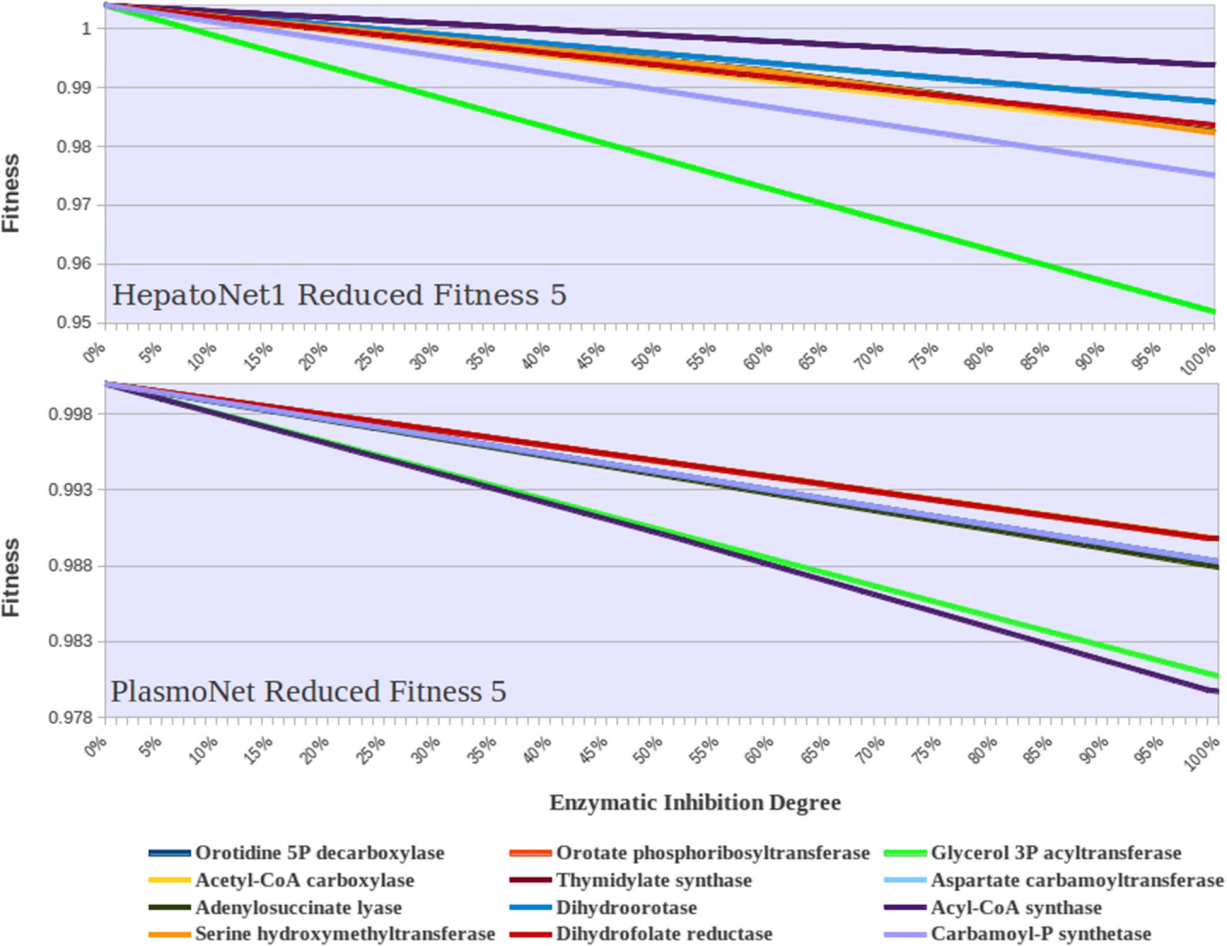
HepatoNet1 and PlasmoNet fitness profiles.

The behavior of the predicted fitness functions, that were simulated for this enzymatic set, are depicted in Figure 1.

### Reduced fitness rank of essential drug targets

For the 12 essential enzymes, we applied the reduced fitness concept to assess in a more subtle way their impact on the two metabolic networks (see Table 1). To validate the outcome of the reduced fitness rank, three enzymes were shortlisted from Table 1: acyl-CoA synthetase (score: 1.97); aspartate carbamoyltransferase (score: 0.713); glycerol-3-phosphate acyltransferase (score: 0.37).

The impairment of *acyl-CoA synthetase* in HepatoNet1 caused a perturbation in the mitochondrial synthesis of cardiolipin (–50%), while in PlasmoNet the inhibition severely destroyed the production of sphingomyelin (–97.07%). HepatoNet1 mitochondrial cardiolipin was produced by the condensation of glycerol-3-phosphate and CDP-diacylglycerol, that was obtained by mitochondrial phosphatidate. This latter metabolite was synthesized from acyl-CoA mitochondrial pool (that collects many activated fatty acids as palmitoyl-CoA and oleoyl-CoA). Cytosolic acyl-CoA syntethase was responsible for the formation of these activated fatty acids, thus an enzymatic impairment can affect the cardiolipin synthesis. This is experimentally demonstrated in human tumoral cells [32]. Acyl-CoA synthetase inhibition impaired PlasmoNet sphingomyelin production: the main precursors of sphingomyelin are serine and palmitoyl-CoA, that was produced in the cytosol by the enzyme. The parasite shows a high activity of fatty acid anabolism, thus the enzyme is important for plasmodial growth [33]. During the intra-erythrocytic stage, the parasite synthesizes new sphingolipids which are necessary for the formation of the tubovesicular network [34]. The formation of this membrane structure connects the parasitic vacuole with the host membrane during invasion. A possible activation of sphingomyelinase, that degrades sphingomyelin pools, provokes the plasmodial death. This suggests that a certain amount of sphingolipids is essential for *Plasmodium falciparum*.

The second enzyme is *aspartate carbamoyltransferase* whose inhibition caused an impairment of UDP-glucose production in HepatoNet1 (–80.57%) and mRNA in PlasmoNet (–57.44%). It is reported that inhibitors of aspartate carbamoyltransferase cause a 10% reduction of the UTP intracellular pools in hepatoma cell culture [35].

The last enzyme is *glycerol-3-phosphate acyltrans- ferase*, that caused dramatic effects in the hepatocyte metabolism, hindering the production of phosphatidylethanolamine (–55.77%), phosphatidylinositol (–50%), phosphatidylcholine (–50%), cardiolipin (–50%) and triglycerides (–50%). Phosphatidate is a common precursor of these metabolites and is formed in the human in the linear chain from 1-acyl-glycerol-3-phosphate, a product of glycerol-3-phosphate acyltransferase. A specific inhibitor of this enzyme (FSG67) causes in obese rats the reduction of triglycerides and phosphatidylcholine [36] and this was also confirmed by our analysis. The *in silico* inhibition of the enzyme impaired the production of phosphatidylcholine (–94.55%) in PlasmoNet. The plasmodial gene sequence that encodes glycerol-3-phosphate acyltransferase is expressed in double yeast mutants and biochemically characterized [37]. The authors suggested that it is likely that glycerol-3-phosphate acyltransferase is essential for a growing *Plasmodium falciparum*, that requires high amount of phospholipids for membrane synthesis. This hypothesis is sustained by another work, whose biochemical analyses show that parasites in trophozoite and schizont stages have an high acyltransferase activity [38].

Although it was not possible to assess the quantitative aspect of the selectivity score with experimental evidence, there was a good agreement among the obtained results and the literature.

### Homologous drug targets predicted non-essential in both networks

Twelve drug targets showed up to be non-essential in both networks (Table 1), although their essentiality is experimentally validated for the parasite. In 8 cases the conflicting outcomes could be sorted out with literature-based assessment of the *in vitro* medium compositions and restriction of indicated inbound fluxes (*conditional essentiality*).

The remaining discrepancies may be due to possible molecular interactions that were not considered in the network reconstruction, e.g. off-target effects. For example, Velanker and coworkers find that plasmodial glycolytic enzymes associate with membranes and cytoskeleton components and drain their substrates near to the invasion machineries, making contact with host microtubules [39]. The authors also suggest that the inhibition of the glycolysis is achieved with enzymatic inhibitors or, alternatively, with the disruption of the cytoskeleton assembly. It is then likely that plasmodial glycolytic enzymes form a multi-enzymatic complex that is associated to the cytoskeleton. This hypothesis is also supposed by an old theoretical work, that suggests that the glycolytic enzymes are not ”evenly distributed throughout the cytosol”, but are likely localized in restricted regions [40]. It is also likely that these cytoskeleton-associated enzymes have an important role during host invasion and, thus, their inhibitors may cause off-target effects. In this set, gene deletions predicted that 3 glycolytic enzymes were dispensable. These were aldolase, hexokinase and triose-phosphate isomerase. Parasitic *aldolase*, for example, binds AMA1 effector to initiate host invasion [41]. Furthermore, the parasite relies on the glycolysis for its own ATP production, that is invested for growth, replication, motility and invasion. *Hexokinase* is found to be essential for the intra-erythrocytic stage of the protozoan [42]. On the other hand, it was predicted that hexokinase in intra-hepatic stage was dispensable for the achievement of the biomass reactions. Then, to simulate the effects of the upper glycolytic branch disruption, a double knock-out of hexokinase and glucose-6-phosphate isomerase was simulated. In this case, the impaired biomass components were phosphatidylinositols. This impairment and its consequences were in agreement with experimental assays, where the inhibition of hexokinase provokes the total disruption of the synthesis of glycophosphatidylinositols [43]. It is then likely that hexokinase and glucose-6-phosphate isomerase belong to the same multienzymatic complex that was proposed by Huebscher [40].

The last enzyme of this little set is *triose-phosphate isomerase*, that is expressed on the membrane of infected erythrocytes where it triggers antibody selection and prolonged hemolytic anemia [39].

Because of its specific extra-metabolic functions (infection/immune system activation), it is very likely that this enzyme is connected to off-target effects.

Among the enzymes that are conditionally essential, *ornithine decarboxylase* was found to be essential under the restriction of polyamines (as putrescine and spermidine) and under the block of agmatinase. This enzyme synthesizes the first polyamine: putrescine. In PlasmoNet, a secondary bypass through agmatinase (EC 3.5.1.53) made this enzyme dispensable. Agmatinase was identified in *Plasmodium berghei* and hypothesized in *Plasmodium falciparum* but never characterized here, so this bypass may not be present. Here, a double enzymatic knock-down was simulated and both enzymes resulted to be essential for PlasmoNet. Recently, it has been reported that a full perturbation of ornithine decarboxylase triggers sophisticated compensatory mechanisms on the transcriptome, proteome and metabolome of the parasite [44]. It is then likely that the enzyme is *per se* essential for the parasite and that the “rescue” mechanism is a evolutionary survival strategy. The literature indicates that the same enzyme is dispensable for the human host, suggesting a similar underlying conserved mechanism of regulation [45]. In the set of conditional essential enzymes, *phosphoethanolamine methyltransferase* was found to become essential under depletion of external choline and phosphatidylcholine. This enzyme had a replenishing function in the phospholipid synthetic pathway, transferring 3 methyl groups on the ethanolamine to form the choline head (in case of choline depletion). In *Plasmodium berghei* it has two different substrates, ethanolamine phosphate and phosphatidylethanolamine [46]. In PlasmoNet only the first reaction was included (ethanolamine-phosphate + 3 methyl-donors → choline-phosphate) and it was not active since phosphatidylcholine and choline were available in the simulated medium. This bypass is important only if choline is missing in the external environment, so to assess its essentiality the choline/phosphatidylcholine transporters were blocked and the missing reaction was added in PlasmoNet. Under these conditions the enzyme was essential. The last enzyme that was detected as dispensable is NADH dehydrogenase. Its metabolic functions can be fulfilled alternatively by Complex *bc*_1_. Inhibitors of *NADH dehydrogenase* (as atovaquone) target the mitochondrial electron transport chain but also affect Complex *bc*_1_[47]. Thus, a double knockout of NADH dehydrogenase/Complex *bc*_1_was applied, whose outcome indicated that the synergistic activity of both enzymes was essential for the parasite.

## Conclusions

To increase the selectivity of an anti-pathogenic drug that could target also host enzymes, a conventional way is to exploit structural differences between homologous proteins. On the other hand, network-based modeling methods are alternative and complementary strategies to assess enzymatic inhibitions. In fact, genome-scale networks can detect enzymes that are likely to be essential and selective. Ideally, these approaches identify potential drug targets that are specific to the parasite.

While this idea may be impressive in its simplicity, the amount of parasite-specific enzymes can be restricted. In fact, out of the merged list of 96 “gold standards” only 18 enzymes are specific to *Plasmodium falciparum* metabolism.

It should also be considered that *Plasmodium falciparum*, when exposed to drug-induced selective pressure, develops drug-resistance (e.g. pyrimethamine and chloroquine) [48]. In this context, it would be nice to have more predictive methods and this was the rationale behind this research. The question this work aimed to address was the assessment of the selectivity of antimalarial drug targets with FBA-based methods. A human hepatocyte metabolic network was chosen to represent the host metabolism since the liver is the first human infection site for malaria parasites [17]. Furthermore, the liver possesses the most versatile metabolism among human cells. In fact, it is likely that drug targets, that compromise the metabolism of any human cell, should also be identifiable in the hepatocyte. Furthermore, it is the preferred organ to investigate the drug-induced metabolic impairments, whose consequences may be not detectable in preclinical and clinical trials [49].

To apply FBA-based methods, the main requirement is the availability of curated genome-scale metabolic networks. Furthermore, to achieve a realistic flux distribution, each model should be simulated with a literature-based set of metabolic objectives. This set allows the simulation of an anabolic physiological state of the metabolic model, in similar way to the biomass objective function [50]. The applied metabolic objective sets were assembled, approximated and rescaled from a multitude of different sources. The relative concentration share of each biomass component was taken as an estimate of its production rate. A physiological assembly of the metabolic objectives was necessary to describe realistically the consequences of the inhibitions of chosen antimalarial drug targets.

Overall, this work predicted that a large drug target set was non-essential for the hepatocyte model (24/48) and that 12 enzymes were instead essential for both models. For the first set gene deletions were simulated, while for the second set the concept of reduced fitness was applied. This last method can analyze more in detail the drug-induced impairments and their consequences on the network performance.

Gene-deletion simulations are a mean to assess the importance of a given biochemical reaction for the functionality of the network. This type of simulations are comparable to experimental gene-excision methods: the enzyme sequence is *a priori* disrupted and the resulting metabolic consequences are then investigated. On the other hand, it is rare that an administrated drug can achieve 100% enzymatic inhibition. To simulate this last scenario, the concept of reduced fitness was applied. In this case, 12 enzymes, that gene deletions predicted to be essential in both models, were studied in the context of reduced fitness. This method allows to compare the relative enzymatic essentiality and to understand which model is more sensitive to a chosen enzymatic restriction. Unfortunately, the predicted selectivity score could not be validated with the available pharmacological data (e.g. drug binding constant for the target and cytotoxic IC50 index) for infected and non-infected hepatocyte cultures. Our work had clearly some limitations, that were due to lacking pharmacological data on antimalarials and approximations of the metabolic objectives. Then, genome-scale metabolic networks do not usually integrate regulatory feed-back loops and, thus, they may be not feasible to predict consequences of enzyme inhibition that are triggered by negative regulation. As mentioned above for the case of thymidylate synthase, enzymatic drug-induced inhibition may cause enzymatic over-expression and this aspect can not be exhaustively predicted with the methods that are here applied.

Despite the applied approximations and the missing data, the obtained results were in agreement with the available literature. Thus, this framework may be useful to detect putative selective drug targets, that gene-deletion simulations may discard. Further analyses on the selectivity of antimalarial targets (by means of RNA interference and covalent inhibitor assays) are therefore required to validate the predicted selectivity score. RNA interference, for example, allows to tune the degree of inhibition, avoiding off-target effects due to unspecific protein binding [51]. Alternatively, enzyme impairments by covalent inhibitor assay [52,53] could provide the experimental mirror of the computed fitness function. In this last case, drug binding constant and cytotoxic index for each inhibitor will be useful to assess the ”pure” network effects that were here predicted. In fact, cytotoxic index alone is not informative in this respect, as it can not discriminate among strong network effects under weak drug binding and weak network effects under strong drug binding. Finally, the last important aspect to consider is the choice of the strain of *Plasmodium falciparum*, as drug resistance and sensitivity may largely vary among different strains. This is the rationale why anti-pathogenic drug discovery is now focusing on drug combinations, that are effective against a wide spectrum of pathogenic strains in low doses [54].

## Methods

### Drug target selection

**Figure 2:**
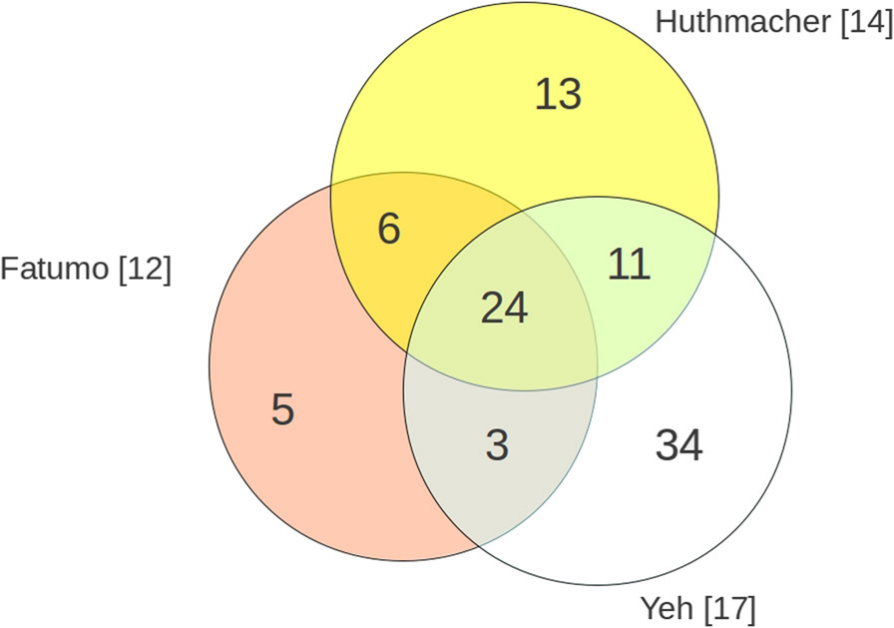
**Antimalarial drug targets and their overlap.** For each circle the name indicates the first author. Globally the scheme depicts the distribution of 96 antimalarial drug targets.

Three literature-based datasets of validated antimalarial targets [14,18,19] were collected, merged and pruned. The merged set contains 96 experimentally validated essential enzymes reported for the parasite *Plasmodium falciparum*. Three enzymes are targets of approved drugs and only two of them are common in all sets (dihydrofolate reductase, dihydropteroate synthase). For 18 targets, no homologous enzymes are present in HepatoNet1. These pathogen-specific enzymes are suitable as drug targets and require no further *in silico* flux-based investigation. Thirty other enzymes belong to genetic functions and are only remotely intertwined with metabolism (e.g histone deacetylase, telomerase). Thus, they were discarded, since they are not represented in the networks. The remaining 48 enzymatic drug targets were present in both models. In Figure 2 the overlapping of the three datasets is depicted; the full list is given in Additional file 1.

### The metabolic models

HepatoNet1 is a primarily literature-based metabolic network of a human hepatocyte, whose reactions are individually curated and functionally tested [16]. It comprises 2539 reactions, 704 genes and 1149 metabolites. To simulate the pathogen, PlasmoNet model was chosen (Biomodels database ID: MODEL1111240000). PlasmoNet is a large metabolic network of *Plasmodium falciparum*, whose reconstruction is based on information from several databases and extensive literature search [14]. While the published version consists of 1622 metabolites, 1375 reactions and 579 genes, here the model was modified for the scope of the research and the current version (PlasmoNet v2.0, Biomodels database id: MODEL1206070000) contained 1394 reactions: 20 inbound reactions, measured with metabolomics assays [55], were included; one reaction of steroid hormone pathway was removed (KEGG id: R01836; EC 1.1.1.239), since it is likely to be present only in mammalian genitourinary system [56]. Previous versions of the KEGG database [57] indicated that this reaction belongs to the plasmodial metabolism. The correction was then introduced in KEGG version 57.0 and above. Furthermore, an irreversible directionality was added for the reactions catalyzed by S-adenosyl-L-methionine decarboxylase (EC 4.1.1.50) and phosphoenolpyruvate carboxylase (EC 4.1.1.31), as indicated in BRENDA database [58]. The PlasmoNet v2.0 is included as Additional file 2. Detailed information about the applied parameters (reaction directions, imported/exported metabolites) is available on request.

### Definition of metabolic objectives

The metabolic objectives of the two networks were formulated in terms of the so-called biomass reactions which: (i) yield building blocks for the cellular composition, (ii) remove potentially harmful metabolites (e.g. toxins) and (iii) are exported by the cell in the context of systemic physiological functions. For HepatoNet1, literature search collected concentration shares of 98 metabolites, that are cellular building blocks or that can be exported into the blood. The list of applied HepatoNet1 metabolic functions is included as Additional file 3. In some cases, human liver data were not available, thus murine information was applied and rescaled. Also several approximations and assumptions were applied (e.g. the amount of cardiolipin is measured in the periportal and perivenous liver areas [59] and in this case an arithmetic mean of the two concentrations was applied). The metabolites, that HepatoNet1 was allowed to import from the extra-cellular environment, were the human blood components and their inbound fluxes were left unconstrained [16]. The set of PlasmoNet biomass components consisted of 98 metabolites, whose concentration shares were retrieved from literature. An initial set of 57 values was already published along with PlasmoNet reconstruction [14], but for the scope of the research this set was enlarged to 98 metabolites. The environment in which PlasmoNet is embedded represented the host cytoplasm. To describe a feasible situation for a growing parasite, all host-pathogen exchange reactions were left unconstrained. The full list of PlasmoNet metabolic objectives is given as Additional file 4.

### Gene deletions

To test the essentiality of drug targets, gene deletions were performed under the flux minimization framework [60], in similar way to previous studies [6-8]. The optimization problem aimed (i) to minimize the sum of internal fluxes and (ii) the simultaneous achievements of all biomass reactions. To simulate gene-deletion of a drug target, the fluxes through the corresponding catalyzed reactions were constrained to zero. Under these conditions, a successful simulation would predict that the enzyme is non-essential and all biomass reactions can be fulfilled. If no feasible solutions can be reached, the enzyme is considered to be essential. A more detailed explanation of the flux minimization problem is given in Additional file 5: Appendix A.

### Reduced fitness approach

This approach has been applied to an erythrocyte model to simulate the impact of enzyme deficiencies on network performance [20]. The initial step is the calculation of a reference state without any impairment. Then, for each potential target enzyme, a metabolic scenario is simulated, where (i) the fluxes through the reactions catalyzed by this enzyme are progressively restricted and (ii) the deviation of the biomass fluxes from the reference state is minimized. The deviations express the model’s impairment under the enzymatic perturbation, the inverse is called *fitness*. A detailed explanation of this concept together with an exemplary application are given in Additional file 6: Appendix B.

### Enzymatic fitness estimation and selectivity score

To assess the network performance under an enzymatic impairment, the initial reference state is obtained with flux minimization method. This framework does not predict a unique solution but this aspect is common to many FBA methods that are based on optimization problems. To calculate the reduced fitness for an enzymatic knock-down, the enzyme-catalyzed null fluxes are blocked. The non-null fluxes, instead, are subjected to a progressive reduction of 1% of the reference flux values for each simulation run. For each decremental step of the non-null fluxes, a single value of reduced fitness is calculated. In this way, a single fitness curve is obtained for each enzymatic knock-down (Figure 1). To define selectivity in terms of the reduced fitness, the area under the fitness curves (AUC) of the networks is calculated and compared for each enzymatic inhibition. In case of a non-essential drug target, the obtained fitness curve is a straight horizontal line at fitness 1 and, applying the progressive decrement of 1% of the reference enzymatic activity, its AUC is 100 (as a per cent measure). The larger the area under the fitness curve (i.e. the smaller its deviation from 100), the less is the effect of the drug target on metabolic fitness. The AUC deviation from 100 defines the selectivity score (equation 1).

(1) AUCdev=100−AUC

Then, the selectivity score of a drug target is defined as

(2) $$\text{Selectivity Score}=\frac{{\text{AUCdev}}_{\text{PlasmoNet}}}{{\text{AUCdev}}_{\text{HepatoNet1}}}$$

Athe possible extra-metabolic role of the biomass components.

## Competing interests

The authors declare that they have no competing interests.

## Authors’ contributions

SB developed the original idea, carried out all computational analyses and drafted the manuscript. AH provided tools for the knock-out simulations and together with HGH and SB participated in the design and evaluation of the analyses. All authors contributed to and approved the final manuscript.

## Supplementary Material

Additional file 1 Merged set of validated antimalarial targets. Click here for file

Additional file 2 **Updated version of PlasmoNet.** The network is available on Biomodels database under the ID MODEL1206070000. Click here for file

Additional file 3 HepatoNet1 applied metabolic objectives. Click here for file

Additional file 4 PlasmoNet applied metabolic objectives. Click here for file

Additional file 5 **Appendix A.** Flux minimization optimization framework. Click here for file

Additional file 6 **Appendix B.** Reduced Fitness optimization framework and example. Click here for file
