# Supplementary material for: Network-based assessment of the selectivity of metabolic drug targets in Plasmodium falciparum with respect to human liver metabolism
Source: BMC Syst Biol. 2012 Aug 31;6:118. doi: 10.1186/1752-0509-6-118 (PMC3543272; doi:10.1186/1752-0509-6-118)
Supplement: Additional file 5 — Appendix A. Flux minimization optimization framework. [file 1752-0509-6-118-S5.pdf]

Appendix A: Flux minimization

Maintenance/Biomass flux

Specific outbound fluxes are defined in this appendix as *metabolic objectives* and are a mean to describe the anabolic performance of the model. Each metabolic objective corresponds to a single maintenance/biomass component that is synthesized and then exported into the environment. Since each component is present *in vivo/in vitro* in a specific concentration share, a literature-based value represents this share in mmol/g·h and is associated to each metabolic objective. Each simulation aims to fulfill all the metabolic objectives. This is the main *discrimen* between a successful computation and a failing one (where one or more target reactions can not be fully satisfied). From a physiological point of view, this set of fluxes depicts the metabolic requirements that the network in a feasible solution should achieve. From a comparison with the biomass objective function that is applied in flux balance biomass maximization, the rationale behind biomass assembly is very similar to the one applied here. The main difference is that, while the biomass maximization predicts the highest achievable value for the biomass production, here the cellular components are demanded to be produced under any condition and their production is not optimized, but kept constant.

Objective Function

As explained above, the objective function here applied is the minimization of the sum of the internal fluxes. The rationale behind is the description of the cellular minimal metabolic efforts in the achievement of physiological functions. The minimal metabolic efforts are here represented by the minimization of the sum of n internal fluxes.

$$\Phi = \sum_{i=1}^{n} |v_i| \tag{1}$$

Gene Deletions

To simulate the impact of an enzymatic knock-out, the set of the reactions that are catalyzed by the same enzyme are *a priori* blocked. This means assigning a null value to these reactions, setting their lower and upper bounds. If the role of the enzyme is essential, one or more of the metabolic objectives are not fulfilled anymore and the result will be an infeasible solution. If the enzyme is instead non essential, the fullfillment of the metabolic objectives can be still achieved with different flux rearrangements.

Optimization formal problem

Here the metabolic network has n reactions and m metabolites. A subset of reactions (f over n) are irreversible. This network has k metabolic objectives and Ξ enzymes. The literature-based concentration share is defined by λ for each metabolic objective. Each enzyme catalyzes ϕ reactions, where $\phi \subseteq n$ reactions.

Summarizing all the equation in a consistent formulation, the problem to solve is:

$$Minimize \;\; \Phi = \sum_{i=1}^{n} |v_i|$$

subject to

$$S \cdot v_{\mathrm{i}} = 0, \quad \forall\, i = 1, \ldots, n; \quad \text{Mass Balance}$$

$$0 \leq |v_{\mathrm{i}}| \leq \infty, \quad \forall\, i = 1, \ldots, f; \quad \text{Irreversible Fluxes}$$

$$v_{\mathrm{i,j}} = 0, \quad \forall\, i = 1, ..., \phi; \forall\, j = 1, \ldots, \Xi; \quad \text{Gene deletion case}$$

$$v_{\mathrm{j}} = \lambda_{\mathrm{j}}, \quad \forall\, j = 1, \ldots, k; \quad \text{Metabolic objectives/Biomass components fluxes}$$

Implementation Notes

The algorithm here explained has been implemented in FASIMU (www.bioinformatics.org/fasimu/). Bash commands were necessary to create and format FASIMU input files and to retrieve the results.
