# Supplementary material for: Network-based assessment of the selectivity of metabolic drug targets in Plasmodium falciparum with respect to human liver metabolism
Source: BMC Syst Biol. 2012 Aug 31;6:118. doi: 10.1186/1752-0509-6-118 (PMC3543272; doi:10.1186/1752-0509-6-118)
Supplement: Additional file 6 — Appendix B. Reduced Fitness optimization framework and example. [file 1752-0509-6-118-S6.pdf]

Appendix B: Reduced Fitness

The analysis estimates the network performance, that is considered as the simultaneous fulfillment of all metabolic objectives, under perturbation. The only difference between the reduced fitness method and the flux minimization lies in the objective function: in the former it is the it is the maximization of the fitness (defined below) while in the latter it is the minimization of the fluxes. The network impairment is quantified by the fitness function.
In this appendix, this method is applied to a simple glycolytic model in aerobic and anaerobic conditions and the fitness outcome is compared with the results obtained with gene-deletions.

Flux Perturbation & Reference State

In this analysis, the applied perturbation is the restriction of chosen fluxes to describe enzymatic impairments. The key point of this analysis is the choice of the network reference flux distribution to assess the enzyme-catalyzed fluxes in an unperturbed metabolic state. For the reference state, the flux minimization method that is explained in Appendix A is applied. All reactions catalyzed by the same enzyme carrying a non zero flux are restricted by a chosen percentage of the reference value, while the null fluxes are instead *a priori* blocked. This is achieved with the setting of the lower and upper bounds. For each flux restriction scenario, a unique value of fitness is then retrieved.

Fitness Objective Function

The optimization aims to maximize the function in equation 1, where L_i is the literature-based concentration share of the i-th biomass component (or *load value*) and v_i is the perturbation-caused new value of the target flux (or the variation of the synthesis of the specific biomass component). The predicted value is a real number, which range spans from 0 (total network impairment) to 1 (full fulfillment of the target fluxes).

$$Fitness = 1 - \frac{1}{k}\sum_{i=1}^{k}\frac{|v_i - L_i|}{|L_i|} \tag{1}$$

Optimization formal problem

The metabolic network comprises n reactions and m metabolites. A subset of reactions (f over n) are irreversible. This network has k metabolic objectives. Here it is considered the simple case of single inhibited enzyme that catalyzes ψ reactions, out of which η are non-null fluxes and χ are null fluxes (thus, ψ=η+χ for this enzyme). δ indicates the degree of the flux restriction (from 100% to 0% of the reference flux) that constrains the inhibited enzyme fluxes, previously calculated with the flux minimization algorithm ($\mathrm{v_{reference}}$).
Thus, the optimization problem becomes:

$$Maximize \;\; Fitness = 1 - \frac{1}{k}\sum_{i=1}^{k}\frac{|v_i - L_i|}{|L_i|}$$

subject to

$$S \cdot v_{\mathrm{i}} = 0, \quad \forall\, i = 1, \ldots, n \quad \text{Mass Balance}$$

$$0 \leq |v_{\mathrm{i}}| \leq \infty, \quad \forall\, i = 1, \ldots, f; \quad \text{Irreversible Fluxes}$$

$$v_{\mathrm{i}} = 0, \quad \forall\, i = 1, \ldots, \chi; \quad \text{blocked zero fluxes of the perturbed enzyme}$$

$$v_{\mathrm{i}} = \delta \cdot v_{\mathrm{reference,i}}, \quad \forall\, i = 1, \ldots, \eta, \forall \delta = 0, \ldots, 1; \quad \text{restricted non-zero fluxes of the perturbed enzyme}$$

$$v_{\mathrm{j}} = \lambda_{\mathrm{j}}, \quad \forall\, j = 1, \ldots, k; \quad \text{Metabolic objectives/Biomass components fluxes}$$

Glycolysis under aerobic and anaerobic conditions

To illustrate the method of reduced fitness, the reduced fitness was applied to a simple glycolytic network (figure 1) under aerobic and anaerobic conditions. For this example, the reaction $\mathrm{v_{lg}}$ (the lumped lower branch on the glycolytic pathway) was progressively inhibited from the 100% of its predicted value in the reference state to 0%. The network performance (the fulfillment of the target fluxes) was assessed and quantified with the fitness function.

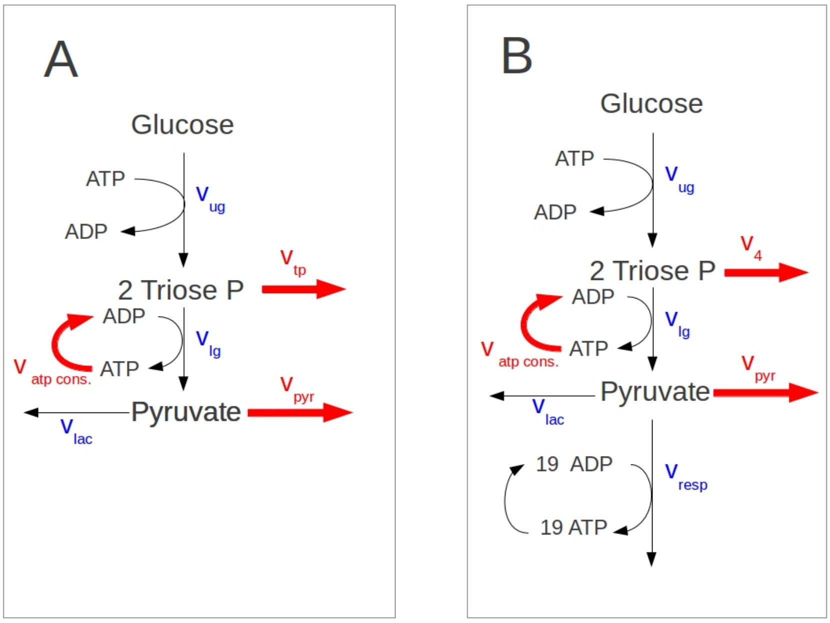

Figure 1: Network Topology of the anaerobic glycolysis (A) and aerobic glycolysis(B). $\mathrm{v_{ug}}$=lumped flux for upper glycolysis; $\mathrm{v_{lg}}$=lumped flux for lower glycolysis; $\mathrm{v_{tp}}$=**metabolic objective** for triose phosphate consumption; $\mathrm{v_{pyr}}$=**metabolic objective** for pyruvate consumption; $\mathrm{v_{atp\ cons.}}$=**metabolic objective** for ATP consumption requirements; $\mathrm{v_{lac}}$=lactate consumption/export; $\mathrm{v_{resp}}$=lumped flux for oxidative phosphorylation

In this model the metabolic objectives are indicated in figure 1 by red arrows and their load values were arbitrarily chosen ($\mathrm{v_{tp}}$=3; $\mathrm{v_{pyr}}$=2; $\mathrm{v_{atp\ cons.}}$=1). $\mathrm{v_{tp}}$ metabolic objective represents the utilization of

dihydroxyacetone phosphate for lipogenesis; v_{pyr} is the drain of pyruvate for the production of Acetyl-CoA; $v_{atp\ cons.}$ depicts the overall ATP demand. Scheme B is an extension of scheme A, as it includes $v_{resp.}$ that stands for oxidative phosphorylation lumped flux, where for each metabolized molecule of pyruvate there is a yield of 19 molecules of ATP.

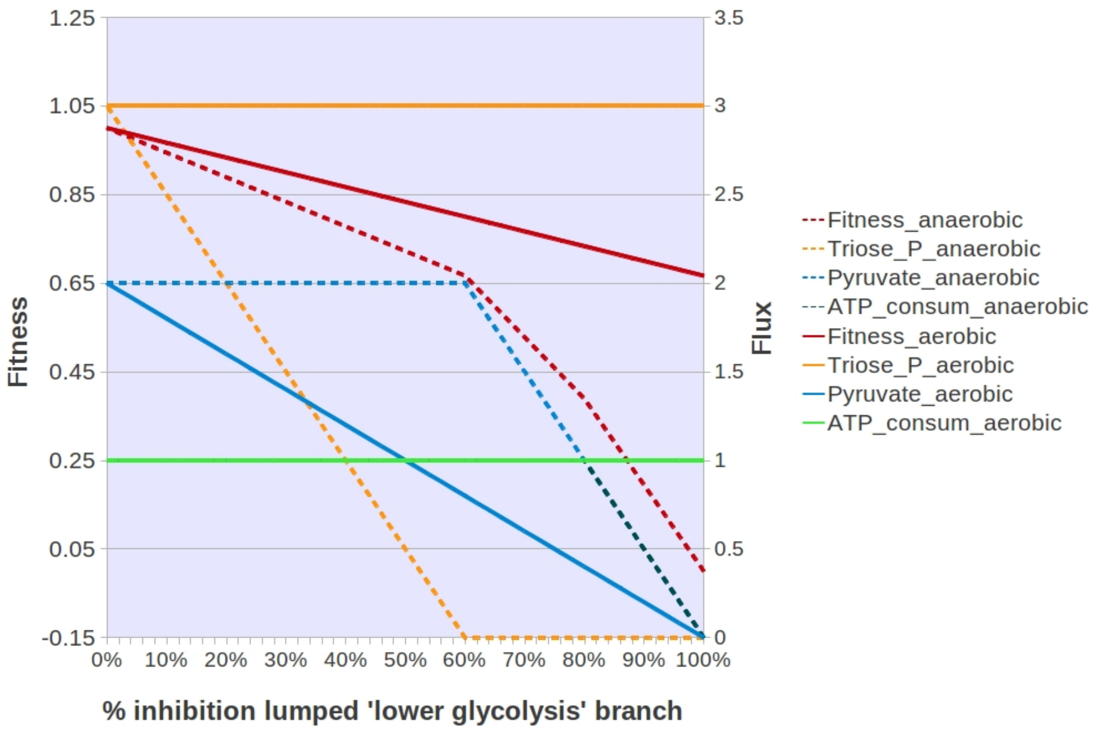

Figure 2: Metabolic Reduced Fitness profiles under the inhibition of v_{lg} flux.

Figure 2 shows the estimated fitness functions for the two models (red lines) and the effects of the inhibition on the metabolic objectives. The fitness of the B model (red continuous line), that is capable of oxidative phosphorylation, handled better v_{lg} perturbation, in comparison with network A fitness (red dotted line); v_{tp} objective of the B model (yellow continuous line) did not variate from its load value (in this case 3) and in this case the restriction of the lower part of the glycolysis did not affect the triose phosphate synthesis in aerobic conditions. Under anaerobic conditions, instead, this metabolic objective was subjected to a severe drop at 60%of v_{lg} inhibition, because of the limited ATP production. The pyruvate metabolic objective of the B model (blue continuous line) was sensitive to v_{lg} perturbation because the inhibition affected directly the pyruvate synthesis and export. The ATP demand, in presence of oxidative phosphorylation (green continuous line), was independent from the applied inhibition. The same metabolic objective in anaerobic conditions (dark green dotted line) was subjected to a severe drop at 82% of inhibition, since the perturbation disturbed the production of ATP required by $v_{resp.}$.

Table 1 reports the results of the gene-deletion approach for each flux of the models. The outcome of

Knocked Out Reaction	Flux Essentiality A	Flux Essentiality B
v_{ug}	essential	essential
v_{lg}	essential	essential
$v_{atp\ cons.}$	essential	non essential
v_{lac}	essential	non-essential
$v_{resp.}$	essential	essential

Table 1: Outcome of reaction knock-out in the A and B models

gene deletions in metabolic networks follows a binary logic (a reaction can be essential or non-essential for the fulfillment of the network requirements) and in this case v_{lg} was essential for B model, while in A all reactions were essential for the fulfillment of the metabolci objectives. The fitness profile of v_{lg} in figure 2 is far more instructive and it is possible to estimate how much a reaction is essential for the models. If the outcome of gene deletions is consistent with a binary logic, the fitness results are more akin to many-valued logic (where the predicted fitness function may range between 0 and 1).

Implementation Notes

The algorithm here explained has been implemented in FASIMU (www.bioinformatics.org/fasimu/), that comes with 8 different fitness functions. Bash commands were necessary to create and format FASIMU input files and to retrieve the results. For automatized routines, bash scripts were implemented.
